# How scaffolds shape MAPK signaling: what we know and opportunities for systems approaches

**DOI:** 10.3389/fphys.2012.00475

**Published:** 2012-12-21

**Authors:** Franziska Witzel, Louise Maddison, Nils Blüthgen

**Affiliations:** ^1^Institute of Pathology, Charité–Universitätsmedizin BerlinBerlin, Germany; ^2^Institute for Theoretical Biology, Humboldt University BerlinBerlin, Germany; ^3^Manchester Institute of Biotechnology, University of ManchesterManchester, UK

**Keywords:** scaffold, signaling, MAPK and ERK signaling, Ste5, mathematical modeling, ultrasensitivity

## Abstract

Scaffolding proteins add a new layer of complexity to the dynamics of cell signaling. Above their basic function to bring several components of a signaling pathway together, recent experimental research has found that scaffolds influence signaling in a much more complex way: scaffolds can exert some catalytic function, influence signaling by allosteric mechanisms, are feedback-regulated, localize signaling activity to distinct regions of the cell or increase pathway fidelity. Here we review experimental and theoretical approaches that address the function of two MAPK scaffolds, Ste5, a scaffold of the yeast mating pathway and KSR1/2, a scaffold of the classical mammalian MAPK signaling pathway. For the yeast scaffold Ste5, detailed mechanistic models have been valuable for the understanding of its function. For scaffolds in mammalian signaling, however, models have been rather generic and sketchy. For example, these models predicted narrow optimal scaffold concentrations, but when revisiting these models by assuming typical concentrations, rather a range of scaffold levels optimally supports signaling. Thus, more realistic models are needed to understand the role of scaffolds in mammalian signal transduction, which opens a big opportunity for systems biology.

## Introduction

The three-tiered MAPK signaling cascade is a highly conserved signaling pathway that regulates the cellular response to a variety of external stimuli in all eukaryotes. A diverse range of receptors initiates this pathway, which consists of a phosphorylation-dependent relay of protein activation, resulting in altered transcription, ultimately regulating processes such as cell proliferation and differentiation. The details of this signaling pathway are very well studied and detailed maps of the function of specific protein kinases and protein phosphatases within the pathway have been produced (Oda et al., 2005). However, in recent years it became clear that a number of scaffold proteins play an essential role in the regulation of this signaling network. Scaffold proteins are defined by the binding of at least two members of a signaling cascade (Chuderland and Seger, 2005; Oda et al., 2005). These scaffolds bring together and organize components of the cascade to facilitate MAPK activation. The proteins that act as scaffolds include KSR1/2, IQGAP1, β-arrestin 1/2, MORG1, MP1, and paxillin in mammalian systems [see Table 1 for an overview, and the website of Rony Seger (http://www.weizmann.ac.il/Biological_Regulation/NewFiles/rony/scaffolds.pdf) for a comprehensive list of scaffolds].

**Table 1:** **Overview of scaffolds for MAPK signaling in mammalian cells and their functions and locations**.

**Scaffold**	**Comments**
KSR1 and 2	Kinase Suppressor of Ras: KSR is bound to MEK in the cytoplasm. Upon Ras activation, KSR translocates with MEK1/2 to the plasma membrane, which brings MEK1/2 in close proximity to its activator C-Raf and downstream effectors of ERK1/2. These interactions result in the formation of the Raf/MEK/ERK complex (Raman et al., 2007).
β-arrestin 1 and 2	β-arrestins are abundant in clathrin coated pits and enhance MAPK signaling by scaffolding C-Raf, MEK, and ERK (DeFea et al., 2000; Shenoy and Lefkowitz, 2011). The β-arrestins act in a similar way to Sef by directing the MAPK signaling to the cytosol and preventing the translocation of ERK to the nucleus.
Paxillin	Located at focal adhesions, alongside other focal adhesion-specific proteins such as Focal Adehsion Kinase (FAK) and actopaxin (Brown and Turner, 2004). Focal adhesions are sites of tight adhesion between the actin cytoskeleton and the extracellular matrix and are regions of signal transduction that relate to growth control. The coordinated assembly and disassembly of protein complexes allows for cytoskeletal remodeling and thus enables cell motility. Paxillin plays a major role within this process by regulating cell spreading and migration (Turner, 2000). Paxillin is constitutively bound to MEK at the focal adhesions and in response to hepatocyte growth factor binds MEK and ERK and then binds FAK which promotes the activation of phosphatidylinositol-3kinase (PI3K) and Rac (King et al., 1998).
MP1	MEK Partner-1/MPKS1 Mitogen-activated protein kinase scaffold 1 MP1 selectively binds to MEK1 and ERK1, but not MEK2 or EKR2 (Schaeffer et al., 1998). By bringing MEK1 and ERK1 in close proximity to one another, MP1 enhances signaling through this pathway. This complex is targeted to late endosomes by the interaction of MP1 with the endosome protein p14 and enhances the MAPK signaling toward this compartment (Wunderlich et al., 2001). Down-regulation of MP1 or p14 expression reduced ERK/MAPK activation, whereas over-expression increased ERK/MAPK signaling (Teis et al., 2002). Furthermore, the increased ERK activation was dependent on the localization of the scaffold complex to the late endosomes as mislocalization of MP1 caused damping of the duration of the MAPK signal. The MP1-p14 scaffold also interacts with PAK1 and results in enhanced phosphorylation and activation of MEK during cell adhesion and spreading on fibronectin (Pullikuth et al., 2005). In addition, MP1 also interacts with MORG1.
MORG1	MAPK organizer MORG1 binds C-Raf, MEK, ERK, and MP1. MORG1 behaves according to the prozone effect. Interestingly, stimulation of cells with serum or lysophosphatidic acid enhanced ERK activity whereas stimulation with EGF did not (Vomastek et al., 2004).
Shoc2/Sur 8	A leucine rich repeat protein which forms a complex with Raf and Ras. Initial studies have shown that when over-expressed, mammalian Shoc2/Sur 8 enhances Raf activation by promoting its interaction with Ras and thus enhancing the strength of ERK signaling (Li et al., 2000). Strikingly, inhibition of Shoc2/Sur8 causes inhibition of MAPK in tumor cells with mutant Ras (Rodriguez-Viciana et al., 2006).
IQGAP1	Binds B-Raf, MEK, and ERK and facilities ERK activation at specific levels of EGF or insulin-like growth factor, which shows that optimal ERK/MAPK activation requires a balanced stoichiometry of the IQGAP1 to signaling proteins (Roy et al., 2004). IQAGP1 is over-expressed in some cancers such as breast and ovarian, therefore its scaffolding functions for the ERK pathway may contribute significantly to tumorigenesis (Jadeski et al., 2008).
PEB1	Phosphatidyl-ethanolamine-binding protein 1/RKIP binds to both Raf and MEK and prevents their physical interaction, inhibiting MEK phosphorylation, and activation by C-Raf and B-Raf (Yeung et al., 1999; Park et al., 2005). Following mitogenic stimulation, RKIP dissociates from Raf to allow MEK activation. RKIP induces switch like behavior of MEK (Shin et al., 2009). It has been demonstrated that RKIP protein expression is down-regulated in various metastatic cancer cells (Granovsky and Rosner, 2008) and is associated with cancer cells becoming resistant to chemotherapy (Keller, 2004; Chatterjee et al., 2004).
Sef	Sef is located at the Golgi apparatus and binds to activated MEK and facilitates activation of ERK. Sef acts as a spatial regulator for MAPK signaling by preventing ERK translocation to the nucleus but retaining it in the cytoplasm. (Torii et al., 2004).

While most systems-biological research into the pathway neglected the presence of the scaffolds, in recent years many detailed mechanistic studies have been conducted that shed light on the function of these proteins. Besides their main function of assembling complexes, scaffolds are thought to minimize crosstalk with other signaling cascades (Dhanasekaran et al., 2007) and likewise mediate crosstalk (Kolch, 2005), protect kinases from phosphatases (Perlson et al., 2006) and target signals to a specific subcellular location (Roskoski, 2012).

Most insights in the function of scaffolds on MAPK signaling have been obtained in studies of MAPK signaling in yeast, where the function of the scaffold Ste5 is well understood. In mammals, the most intensively researched scaffolds are the KSR family members. Within this paper, we will thus focus on describing the different implications of these two scaffolds on the dynamics of the signaling systems, and will revisit mathematical models that have been utilized to study the function of scaffolds in general in the light of data on MAPK signaling.

## Ste5 and KSR—two scaffolds for MAPK signaling with no sequence similarity

Evidence for scaffold proteins in MAPK first came from studies within the budding yeast *S. Cerevisiae*. These investigations showed that Ste5 is critically important for the mating pathway in yeast as mutation of Ste5 resulted in a lack of response to pheromone (Choi et al., 1994; Marcus et al., 1994). Ste5 is involved in assembling the three-tiered cascade consisting of MAP3K Ste11, MAP2K Ste7, MAPK Fus3 through cooperative binding (Flatauer et al., 2005). Although the Ste5 protein in yeast and the KSR protein family in mammalian cells share similar functions as scaffold they lack any sequence similarity. KSR, Kinase Suppressor of Ras, was originally identified as a regulator of Ras following genetic screens in *D. melanogaster* and *C. elegans* (Kornfeld et al., 1995; Sundaram and Han, 1995; Therrien et al., 1995). Mammalian genomes and *C. elegans* have two isoforms, KSR1 and KSR2.

KSR proteins contain five conserved domains, CA1 to CA5. CA1, which is an N-terminal 40-residue region unique to KSR proteins and CA2, a proline rich region, are of unknown function. CA3 is a cysteine rich zinc finger domain, and is essential for membrane localization following activation of the pathway and similar to the corresponding domain found in Raf (Michaud et al., 1997; Zhou et al., 2002). CA4 is a serine/threonine rich region that serves as a binding site for ERK and is also similar to the analogous position in Raf, except that it does not contain a Ras binding domain found in Raf. Located at the C-terminal end is the CA5 putative kinase domain that binds to Raf and MEK and is also closely related to that region in Raf. Because subdomain II of CA5 lacks a catalytic lysine thought to be important for kinase function of the domain (Therrien et al., 1995), there has been much speculation as whether these kinases were catalytically inert. However, in 2011 KSR was shown to have some catalytic activity toward MEK (Brennan et al., 2011; Hu et al., 2011).

**Figure 1:**
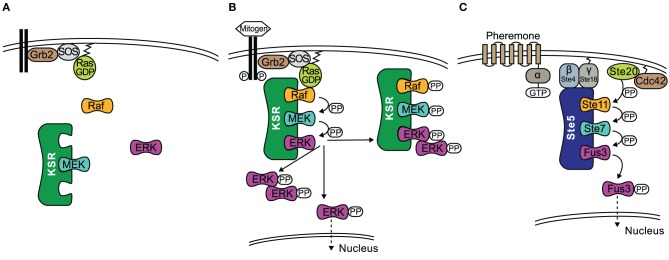
**The role of scaffolds KSR and Ste5 in MAPK signaling. (A)** In quiescent cells an inactive KSR/MEK complex exists in the cytosol. **(B)** Upon stimulation of the cell, KSR translocates to the cell membrane and forms an active complex with phosphorylated Raf, MEK, ERK. Activated ERK detaches from the scaffold with three outcomes; (1) ERK dimerizes in the cytoplasm where the dimer remains or translocates to the nucleus; (2) ERK translocates to the nucleus; (3) KSR acts as a platform where ERK dimers are assembled and the new complex can interact with substrates in the cytoplasm. **(C)** Schematic of the yeast mating pheromone response pathway.

KSR interacts with, B-Raf, C-Raf, MEK1/2, and ERK1/2 coordinating the assembly of these components into a multiprotein complex (Therrien et al., 1996; McKay et al., 2009). In resting cells, MEK1/2 is constitutively bound to KSR1 in the cytosol (Yu et al., 1998), see also Figure 1A. In addition, regulatory 14-3-3 proteins are also bound to KSR at two phosphoserine residues (S297 and S392) and these play a role in localizing KSR in the cytoplasm (Müller et al., 2001). Upon stimulation of the cell, S392 is dephosphorylated by protein phosphatase-2A (PP2A) (Ory et al., 2003), which releases 14-3-3 proteins and allows KSR to re-localize to the cell membrane (Figure 1B), similarly to how 14-3-3 regulate the cellular location of the Raf proteins (Jaumot and Hancock, 2001). Once at the cell membrane, KSR interacts with Raf, which is facilitated by the protein connector enhancer of RAS1 (CNKSR1). An effector of KSR1 is the kinase CK2 (casein kinase 2) which has been shown to bind to the scaffold and to maximally enhance growth factor-induced phosphorylation of B-Raf and C-Raf (Ritt et al., 2007). Once Raf is activated, it in turn phosphorylates and activates MEK. At the same time ERK1/2 is recruited and phosphorylated. Subsequently, activated ERK1/2 is released and translocated to the cytoplasm or the nucleus (Figure 1B). It has been shown that both KSR1 and B-Raf are feedback phosphorylation targets of ERK and that this process is enhanced by the docking of activated ERK (McKay et al., 2009). Consequently, the signaling complex is disrupted and thus signaling via ERK is terminated.

Similar to KSR, Ste5 relocalizes to the membrane upon stimulation by a mechanism that is described in the following and visualized in Figure 1C. Receptor activation by pheromone binding is relayed to a trimeric G-protein, which releases its βγ-subunit as a consequence. Gβγ consists of Ste18, which sits at the membrane due to its lipid anchor, and Ste4, which directly interacts with several downstream effectors. Ste4 interacts with Ste20, a p-21 activated protein kinase (PAK) which is itself recruited to the membrane and activated by binding to Rho-like G protein Cdc42. However, Ste4 also binds to the Ste5-Ste11 complex and thus fulfils the task of making Ste11 accessible to phosphorylation by Ste20. Furthermore, Ste5 undergoes some conformational change that leads to oligomerization of the scaffold (Feng et al., 1998; Wang and Elion, 2003; Bhattacharyya et al., 2006). Once Ste11 is active, the signal is transmitted to the scaffold bound kinases Ste7 and Fus3. It has been shown that phosphorylation of Ste7 and ultimately Fus3 strictly requires membrane localization of Ste5, Ste5 binding to Gβγ and the self-interaction of the scaffold Ste5 (Pryciak and Huntress, 1998; Maeder et al., 2007). While there is in general only a weak interaction of Ste5 and Fus3, artificial dimerization of Ste5 stimulated the association of Fus3 (Slaughter et al., 2007). Ste5 also allosterically stimulates the autophosphorylation of Fus3 on Tyr182. Fus3 on the other hand feedback-phosphorylates Ste5, which downregulates pathway output by an unknown mechanism (Oda et al., 2005; Bhattacharyya et al., 2006). For a more detailed description of the pathway we refer the reader to the review by Bardwell (2004).

## Improved signaling performance by the action of scaffolds

How does the presence of scaffolds change the fidelity of signaling? When kinases are bound to scaffold proteins their local concentration is increased. Consequently, targets of the kinases do experience higher kinase concentrations when bound to the same scaffold and thus their interactions are re-enforced (Park et al., 2003; Lamson et al., 2006). For example, activation of MEK is increased by ~10-fold upon KSR1 expression (McKay et al., 2009). Due to the increased contact between kinases and their substrates, scaffolds are primarily thought to enable signaling, or be positive regulators of signaling. Especially weaker signals are believed to be transduced only in the presence of scaffolds, as dynamic relocalization of the scaffold from the cytoplasm to the receptor at the membrane increases the chance of successful signal initiation (Harding et al., 2005). Additionally it has been proposed that scaffolds might protect kinases from dephosphorylation whilst they are bound in the complex (Levchenko et al., 2000). This might be because the phosphatases are freely diffusing and thus are present in much lower local concentrations, or phosphatases might be sterically hindered, though this has not been shown experimentally yet.

**Figure 2:**
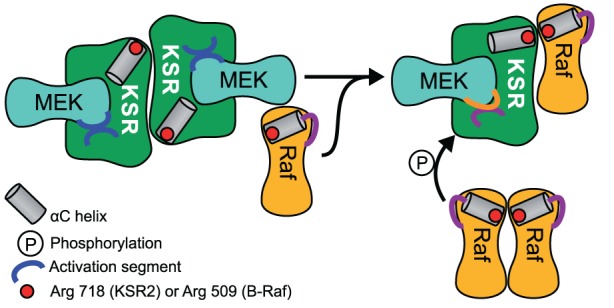
**Schematic of MEK phosphorylation by the allosteric transition of KSR induced by B-Raf binding.** A KSR2-MEK side on side dimer is formed where the activation segments are facing each other and are thus inaccessible. A regulatory Raf interacts with KSR in *cis* to induce a conformational shift of KSR2 α C helix into the active position. Due to steric hindrance a separate catalytic Raf in *trans* can then phosphorylate MEK.

Another way in which scaffolds can contribute positively to signal transmission is by acting as allosteric stimulators. An experimental study in *Drosphilia* Schneider S2 cells observed that the overexpression of KSR increased the amount of active Raf (Udell et al., 2011). KSR allosterically activated the kinase domain of Raf in direct proportion with KSR concentration. How is that possible? The intricate mechanism by which KSR transmits the signal from Raf to MEK was recently revealed in the studies of the crystal structure of MEK bound to KSR2, a homolog of KSR1 (Brennan et al., 2011). Structural analysis and several *in vitro* assays suggest that Raf interacts with KSR in *cis* to induce a conformational switch of MEK. This switch involves phosphorylation of MEK by KSR2 on specific sites that are distinct from the activation segment sites S218/S222. As a consequence MEK exposes its activation segment which is now accessible for a catalytic Raf to interact in *trans* (Figure 2). Similarly, the yeast scaffold Ste5 has a domain that catalytically activates Fus3 for phosphorylation by Ste7 (Good et al., 2009).

## What is the optimal scaffold concentration for best signal fidelity?

The capability of a scaffold to enhance signaling depends on the scaffold's expression level, and both theoretical and experimental analyses suggest that there is a biphasic dependence of scaffold performance on concentration, highlighted also by numerous reviews (Yu et al., 1998; Cacace et al., 1999; Burack and Shaw, 2000; Ferrell, 2000; Levchenko et al., 2000; Heinrich et al., 2002; Kortum and Lewis, 2004; Shao et al., 2006; Ramos, 2008; Chapman and Asthagiri, 2009; Good et al., 2011). Whereas at lower concentrations increasing scaffold concentration facilitates signal transmission, a further increase beyond an optimal level attenuates signaling. Mechanistically, a very high concentration of scaffolds would sequester binding partners in complexes that would be incomplete. For example, a scaffold that brings together MEK and ERK would at high expression levels form mainly complexes with only MEK or only ERK, but only few complexes with both MEK and ERK. This effect is generally known as the prozone effect (Bray and Lay, 1997) and for scaffolds has been termed “combinatorial inhibition” (Levchenko et al., 2000). Theoretical analyses of Heinrich et al. and Levchenko et al. predicted that the optimal scaffold concentration would be in the same order of magnitude as the concentration of interacting signaling proteins (Levchenko et al., 2000; Heinrich et al., 2002).

Such biphasic response has been observed experimentally in different systems. When yeast cells are engineered to express different levels of myc-tagged Ste5, the response of pFus3, pKss1, and a reporter gene is biphasic (Chapman and Asthagiri, 2009). Similarly, when varying the levels of KSR1 in MEFs (mouse embryonic fibroblasts), an optimal level of scaffold exists at which maximal signaling output is observed, both at the level of ERK phosphorylation but also at the level of proliferative and oncogenic capacity (Kortum and Lewis, 2004). While the overall behavior is in line with mathematical models, quantification of the complexes reveals somewhat surprising results: only 5% of the kinases co-precipitate with the scaffold, whereas models would suggest that most kinases would attach to the scaffold. One way to explain this discrepancy is that KSR1 facilitates signaling only in a certain subcellular location, thus effectively having only access to a small pool of the signaling molecules.

## A wide range of scaffold concentrations may optimally support signaling

**Figure 3:**
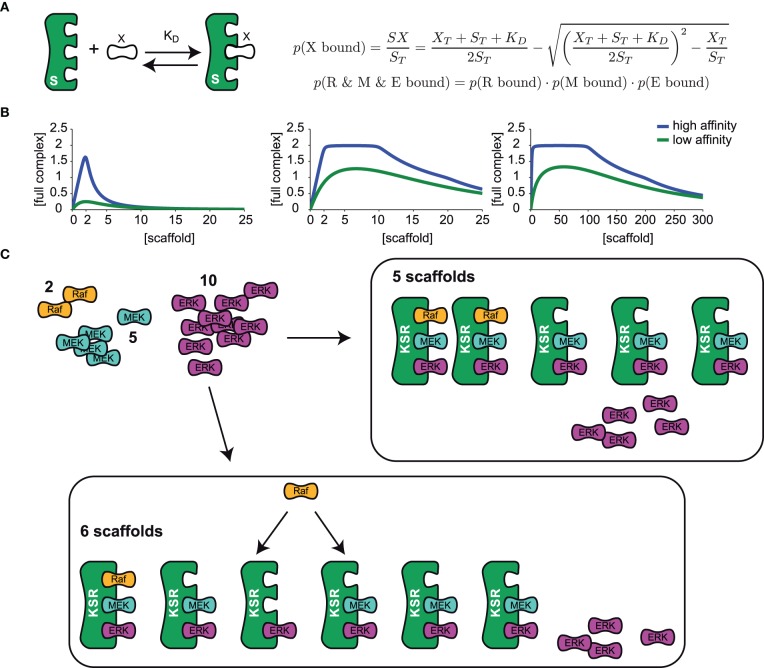
**The optimal scaffold concentration range. (A)** A generic scaffold molecule with three binding sites binds to a kinase X reversibly with the dissociation constant K_D_. The probability for kinase X to be bound to the scaffold is given by the concentration of the scaffold-kinase complex SX divided by the total amount of scaffold S_T_. Assuming that binding of all 3 kinases R (Raf), M (MEK), and E (ERK) is independent from the occupation of the other binding sites, single binding probabilities can be multiplied to obtain the probability for the situation where several kinases are bound at the same time. **(B)** The model presented in **(A)** was used to calculate the concentration of a full scaffold complex (all 3 kinases bound) vs. total scaffold concentration. We consider either an equal amount of ligands (2 Raf, 2 MEK, and 2 ERK, left panel) or different amounts of ligands (2 Raf, 10 MEK, and 20 ERK in the mid panel, 2 Raf, 100 MEK, and 200 ERK in the right panel) for high (K_D_ = 0.01 left and mid panel, K_D_ = 0.1 right panel) and low (K_D_ = 1 left and mid panel, K_D_ = 10 right panel) binding affinity. **(C)** Given a set of various amounts of ligands (here 2 Raf, 5 MEK, and 10 ERK) the optimal range of scaffolds can be explained as follows: Firstly, the least abundant ligand (Raf) determines the maximal number of full complexes that can be formed (two). Until an amount of scaffolds that equals the number of the second less abundant ligand, MEK (5), every scaffold can be occupied with MEK and ERK and so 2 out of 2 possible full complexes can be formed. Having one scaffold molecule more than MEK molecules (6 scaffolds), one scaffold will miss MEK and with a certain probability (*p* = 1/6) Raf will bind to the one that misses MEK and in this case, only 1 out of 2 full scaffold complexes can be formed.

Mathematical analysis so far has highlighted that there is a clear optimum at which scaffolds enhance signaling, and even small deviations from this optimum will lead to attenuated signaling (Levchenko et al., 2000). Considering the natural stochasticity and variability of gene expression (Sigal et al., 2006), it seems unlikely that the cell depends on a strictly regulated concentration of scaffold. For this reason we revisited a previous theoretical analysis that led to the conclusion of an optimal scaffold concentration. We considered a generic scaffold molecule that can bind three kinases. Such scaffold may facilitate signaling when it binds all three kinases at the same time, but it could attenuate signaling when it binds only one or two kinases. For simplicity we made the assumption that binding of the ligands to the scaffold happens independently from each other. Under these assumptions, one can then use occupation probabilities to calculate how many scaffolds carry complete kinase cascades and thus support signaling (Yang and Hlavacek, 2011): when a ligand X is in a dynamic equilibrium with the scaffold S (see sketch in Figure 3A), we can calculate the concentration of the scaffold-ligand complex denoted as SX from the following mass-balance equation:

$$\frac{d\text{SX}}{dt}={\text{k}}_{\text{on}}\;\times S\;\times X-k_{\text{off}}\times \text{SX}$$

When we relate S and X to the total amounts of scaffold S_T_ and ligand X_T_ and replace the ratio of k_off_/k_on_ with the dissociation constant K_D_, we find

$$\frac{\left({\text{S}}_{T}-\text{SX})\times ({\text{X}}_{T}-\text{SX}\right)}{\text{SX}}=K_{D}$$

This can be solved for SX, as shown in Figure 3A. The occupation probability *p*(X bound) is given by the ratio of the concentration of the complex SX and the total scaffold concentration S_T_. Because we assume that binding occurs independently, the probability that all three ligands are bound at the same time is simply given by the product of the single binding probabilities as shown in Figure 3A.

Figure 3B shows the concentration of the fully occupied scaffold complex vs. the total concentration of the scaffold for different binding affinities of the kinases, one for a high and one for a low (yet equal) binding affinity of all kinases. When the concentration of the three kinases is identical, there is a sharp optimum of scaffold concentration exactly at the level of the ligands (Figure 3B, left panel). Interestingly, when the kinases are available at different concentrations, there is an optimal range of scaffold concentrations framed by the level of the least frequent and the 2nd less abundant kinase (Figure 3B, middle panel). In mammalian cells, kinases in the MAPK signal transduction pathways are present in very different concentrations, as Raf is typically present in a very low concentration (~13 nM in HeLa cells), (Fujioka et al., 2006) while MEK and ERK are rather present at concentrations of about 1 μM (also in HeLa cells) (Aoki et al., 2011), thus the concentration ratio of Raf and MEK is at around 1:100. Given this difference in ligand concentration, a 100 fold change in scaffold expression would not change its ability to optimally support signaling, which is illustrated in the right panel of Figure 3B. In line with this model, single cell measurements of ERK phosphorylation in Jurkat cells have confirmed that activation of ERK was unchanged until 100 fold overexpression of KSR1 (Lin et al., 2009). Surprisingly, a recent quantification of the proteome of HeLa cells unveiled that KSR1 is expressed with 1800 copies per cell, which is about 3-fold lower than the expression of Raf and more than an order of magnitude lower than the expression of MEK or ERK in these cells (Nagaraj et al., 2011). This suggests that KSR1 may have an regulatory impact in these cells, as we predict that upregulation of KSR1 will enhance signaling.

## An intuitive explanation for an optimal scaffold range

There is a straightforward and very intuitive explanation for the results of the mathematical treatment. Consider a set of 2 Raf, 5 MEK, and 10 ERK molecules (as shown in Figure 3C) that bind to a scaffold very tightly with a very low dissociation constant (~0), the model predicts an optimal scaffold number between 2 and 5. This is because firstly, the least abundant component, which is Raf in this example, determines the maximal number of full complexes. At least the same number of scaffold molecules has to be available to actually form the maximum number of full complexes (2). When the number of scaffold molecules is in accordance with the level of MEK (5), every scaffold can be occupied by MEK. Also, every scaffold can bind ERK as this component is in excess. The 2 Raf molecules will bind to two arbitrary scaffold molecules and thus the optimum number of full scaffolds is reached (see Figure 3C). However, when the number of scaffolds starts to exceed the level of MEK by one molecule, one Raf molecule may either bind to the scaffold containing MEK and ERK or to the scaffold that misses MEK (Figure 3C). In the latter case only one fully occupied scaffold will be formed. That is why the concentration of a full scaffold decreases when the scaffold concentration exceeds the level of MEK. The exact position of the optimal scaffold concentration range depends on the dissociation constant of the single kinases. With higher dissociation rates the chance for a kinase to be bound to the scaffold decreases and thus the optimal scaffold concentration range is curtailed (compare the curves for high and low dissociation rates in Figure 3B).

## The assembly of scaffolding complexes is highly dynamic

The function of scaffolds critically depends on tight binding of the pathway kinases. However, not all MAPK components have the same affinity. The interaction of KSR1 and MEK has even been shown to be constitutive (Denouel-Galy et al., 1998; Stewart et al., 1999). Tightly bound kinases are restricted in their diffusion away from the protein complex which limits their turnover and spatial redistribution, thus signal amplification might be reduced (Good et al., 2011). Contrary the terminal kinase would have to be bound rather loosely to explore targets within the cytoplasm and the nucleus after its activation. A solution to the paradox is a dynamic regulation of the binding affinity of the terminal kinase to the scaffold. A prototype example for such substrate release is the MAPK Fus3, which has been shown to bind relatively weakly to Ste5 and is easily released allowing signal amplification and also translocation to the nucleus (Good et al., 2009). The release of Fus3 is promoted by the dephosphorylation of several distinct sites at Ste5 by the pheromone-induced phosphatase Ptc1. Dephosphorylation of these sites decreases the binding affinity of the Ste5-Fus3 complex, allowing release of activated Fus3 (Malleshaiah et al., 2010). In accordance, Bhattacharyya et al. found that when replacing wild type Ste5 with a Ste5 mutant of impaired Fus3 docking, expression of a Fus1-GFP reporter increased 2-fold (Bhattacharyya et al., 2006). The authors conclude that the normal function of Ste5-Fus3 docking might even be to attenuate signaling to some degree.

Also, the ternary interaction of the scaffold KSR1 with B-Raf and MEK is highly dynamic. Stimulation of cells with EGF loads B-Raf to the scaffold, also ERK is localized to this complex and phosphorylated thereafter. When docking of ERK is impaired by a mutation, the KSR1-MEK-B-Raf complex persists even 20 min after stimulation, where otherwise, almost all B-Raf has left the complex at this time. However, even when ERK-docking is impaired, it is still phosphorylated. Both KSR1 and B-Raf are feedback phosphorylation targets of ERK and these feedbacks are enhanced by docking of ERK (McKay et al., 2009). This feedback phosphorylation seems to be related to the dissociation of the signaling complex, as ERK-mediated feedback phosphorylation is shown to prevent sustained ERK signaling (Canal et al., 2011).

## Scaffolds might impose stoichiometric restrictions

**Figure 4:**
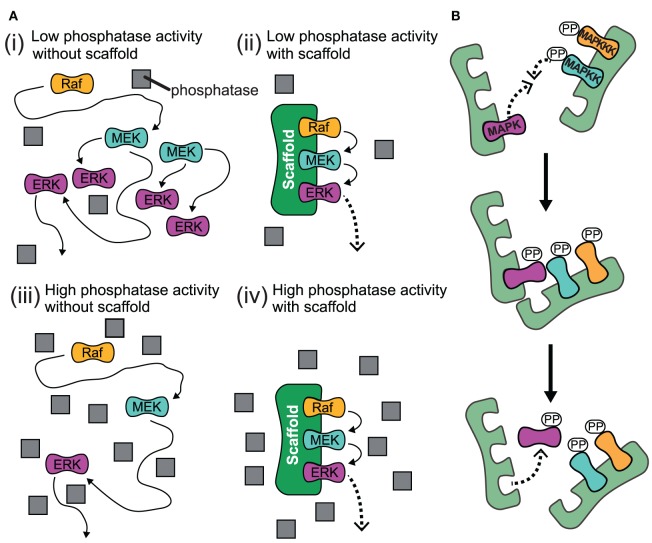
**Proposed effects for the action of scaffolds. (A)** Schematic for high and low phosphatase activity with and without scaffold. (i) When there is low phosphatase activity and no scaffold present, the kinases can readily activate each other and the signal spreads exponentially along the cascade. (ii) When kinases are bound to scaffold under low phosphatase activity, each kinase is localized so it is only likely to interact with their neighboring substrate kinase and signal amplification is attenuated. (iii) When the level of phosphatase activity is high and there is no scaffold for kinases to complex with, the propogation of the signal is impeded as the kinase will likely encounter a phosphatase and become deactivated before it meets its downstream target. (iv) Under conditions of high phosphatase activity, the scaffold complex facilitates signal amplification due to the enhanced local concentration of kinases. This is because the likelihood for a kinase to successfully interact with its substrate kinase is much higher than for the encounter with a deactivating phosphatase which is not localized to the scaffold. Gray squares represent phosphatases. **(B)** *Trans* activation of incomplete scaffold complexes. Kinases bound to different partially occupied scaffolds may activate each other in *trans*.

The experimental studies that we have discussed so far summarize how scaffolds enhance signaling. A recent theoretical study highlights that scaffolds can have dual effects on signaling: Either they support or they attenuate signaling depending on the level of the generic phosphatases as shown in Figure 4A (Locasale et al., 2007). When kinases activate each other in solution, one kinase can activate multiple targets and thus the signal can spread exponentially along the cascade [case (i) in Figure 4A]. However, when loaded on a scaffold, each kinase can only interact with the neighboring substrate kinase, which prevents strong signal amplification [case (ii) in Figure 4A]. This mechanism is termed stoichiometric inhibition, as the interacting kinases are coupled in a 1:1 ratio when they are bound to a scaffold (Locasale et al., 2007). However, when the level of phosphatases is high, the enhanced local concentrations will lead to signal amplification, as the chance for a successful encounter of the kinase with its downstream kinase is much higher than for the encounter with the deactivating phosphatase which is not localized to the scaffold [case (iv) in Figure 4A]. Thus, scaffolds may provide a mechanism to somehow isolate signaling pathways from changing levels of generic phosphatases: a signaling pathway that consists of freely diffusing kinases may be strongly attenuated when phosphatases are upregulated [case (iii) in Figure 4A], while signaling on scaffolds may be only weakly effected. Cells may utilize this mechanism to vary amplification at some signaling pathways in different contexts or cell types, whereas amplification for those on scaffolds remain unaffected.

## Signaling specificity through scaffolds

The main task of the signaling network is to reliably transduce information and ultimately lead to an appropriate response after receiving a certain stimulus. Thus, robust signaling is important in the sense that the signal does not spread over the entire network, but rather remains confined to the pathways that trigger the desired response to the signal. Signaling pathways often share components such as MAP3Ks, MAP2Ks and adapter molecules, or signal via different isoforms that have high sequence similarity, which for example interact via very similar binding sites. Thus, signals may leak to pathways with other function, leading to undesired crosstalk. Alongside mechanisms of mutual pathway inhibition e.g., by feedback regulation (Nakakuki et al., 2010), and compartmentalization, scaffolds are thought to play a major role in conferring signaling specificity to pathways (Bardwell et al., 2007).

**Figure 5:**
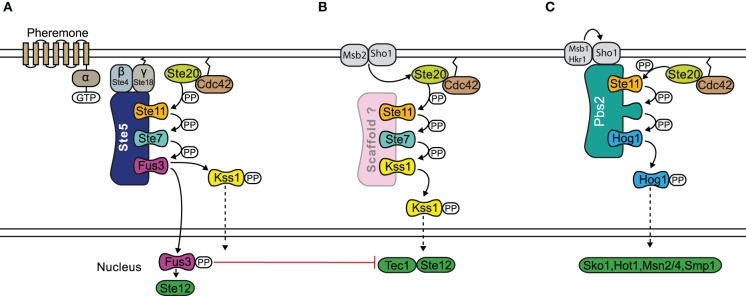
**Scaffold proteins and specificity in the yeast MAPK pathway.** The mating response **(A)**, filamentous growth response **(B)**, and osmotic stress response **(C)** pathways all share the MAP3K Ste11. In the osmotic stress response, Pbs2 acts as a scaffold and also a MAP2K of the pathway at the same time. The mating response and filamentous growth pathways both share MAP2K Ste11 and MAP3K Ste7. Specificity occurs through the scaffold Ste5, which is required for the activation of Fus3 during the mating response. However there may be accidental activation of Kss1 during the mating response and further specification can be achieved by a negative feedback. The filamentous growth pathway possibly does not require a scaffold. Signal flow is shown by black arrows and red T shaped bar indicates inhibition.

Mechanistically, signal specificity may be realized by tethering components to a scaffold by which the components are physically isolated from becoming involved with other pathways (Levchenko et al., 2000). However, such isolation may only work when the rate of deactivation exceeds the rate at which the component moves on and off the scaffold, as otherwise the kinases may move on to different locations, and signaling specificity is lost (Bardwell et al., 2007). How scaffolds confer signal specificity to MAPK signaling is best understood in yeast, where various signaling pathways use joint components (see Figure 5). For example, the mating response, the osmotic stress response as well as the invasive growth response are all transmitted via the activation of the kinase Ste11, a MAP3K. However, the different MAPKs further downstream are selectively activated depending on the stimulus. In osmotic stress signaling, specificity supposedly is achieved by receptor specific recruitment and activation of Pbs2, which is not only a scaffold but also a MAP2K of the pathway at the same time (O'Rourke et al., 2002), which leads to selective activation of Hog1. The mating pathway and the pathway triggering filamentous growth share two kinases: Ste11, a MAP3K and Ste7, a MAP2K. The interplay of various mechanisms allows yeast cells to distinguish between these two conflictive signals: Kss1, the MAPK involved in the invasive growth response, is activated under both conditions, and possibly requires no scaffold. In contrast, Fus3 is only highly activated when the mating pathway is triggered. Mechanistically, strong activation of Fus3 requires the scaffold Ste5, which binds Fus3 with a higher affinity than Kss1 (Choi et al., 1994; Kusari et al., 2004). When binding to Fus3, Ste5 catalytically activates Fus3 for phosphorylation by Ste7 by selectively increasing the k_cat_ for this process (Good et al., 2009). Furthermore, Ste5 itself is thought to be in a closed conformation without pheromone stimulation. In that sense, activation of Fus3 can be understood as a mating-specific phenomenon that is established by selective activation of the scaffold Ste5. Bardwell et al. describe this mechanism of signaling specificity as a logical AND-gate, since it requires activation of Ste5 and the Fus3 (Bardwell, 2006). However, the scaffold obviously cannot prevent accidental activation of Kss1 during the mating response, so that additional mechanisms are required. Although Kss1 is somewhat activated by mating signals, target genes of Kss1 which are typical for the invasive growth response, remain silent. At least two negative paths from Fus3 to Kss1 have been reported and they might act to suppress the Kss1 response when Fus3 is activated, termed cross-pathway inhibition. Thus, in this case, the scaffold generates some signaling specificity, yet downstream cross-talks and feedbacks act to further increase the specificity.

Also in mammalian cells the involvement of different scaffolds can control the dynamics and cellular outcome when the same pathway is activated. For example, when ERK activation is initiated at the cell membrane it is transient and ERK has the ability to translocate to the nucleus. However, the MAPK cascade sequestered at the endosome by the presence of β-arrestin is characterized by sustained activation, leading potentially to the stabilization of transcription factors such as FOSL1 (Nakakuki et al., 2010) and the expression of different genes and more localized ERK activation in the cytoplasm (Kholodenko et al., 2010).

## KSRs in mammalian cells: scaffolds that can route signals to different subcellular locations

**Figure 6:**
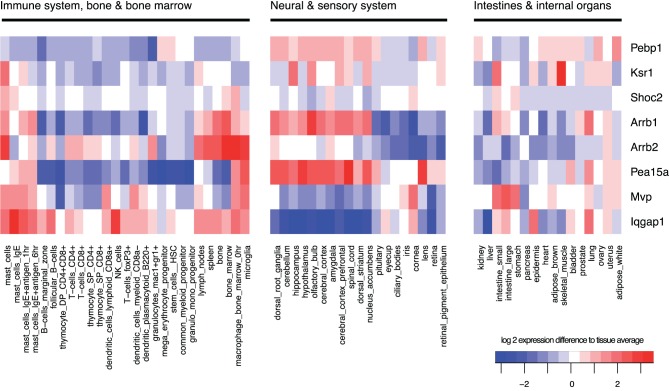
**Tissue-specificity of gene expression.** Gene expression of scaffolds varies strongly between tissue types and each tissue has a specific pattern of expressed scaffolds. Expression was averaged across all samples, and log2-changes from this average are shown in the heatmap, with red indicating high expression and blue low expression. Data were obtained from the GNF mouse gene atlas V3, GEO accession number GSE10246, normalized using rma with standard parameters.

Also in mammalian cells, the MAPK pathway via the terminal kinase ERK triggers various and often opposing cellular outcomes, which include proliferation, apotosis, migration, and cell differentiation. It is however less well-understood how scaffolds contribute to signal specificity, but it is believed that one mechanism is that the interaction of kinases with the large number of different scaffold proteins somewhat determines specificity by targeting the kinases to certain regions of the cell (see Table 1), and by differential expression in different tissues (see Figure 6), the outcome of MAPK signaling might differ strongly. Thus, scaffolds do not only provide a platform on which signaling can happen, but also have been shown to regulate the spatial and temporal signaling of MAPK by directing the signal to specific subcellular compartments (Brown and Sacks, 2009). For example, β-arrestin 2 mainly directs ERK1/2 to the clathrin coated pits (Shenoy and Lefkowitz, 2003) and Sef captures activated ERK at the Golgi preventing nuclear translocation but allowing phosphorylation of substrates in the cytoplasm (Torii et al., 2004). Another example is KSR1 that can translocate dynamically: In resting cells, KSR1 is sequestered by the cytoplasm. Upon stimulation KSR1 translocates to the membrane of the cell to facilitate MAPK signaling, by bringing MEK in close contact to its kinase Raf (Müller et al., 2003). In the brain, where KSR1 is highly expressed, feedback phosphorylation of ERK1/2 regulates the distribution of KSR1 in the post-synaptic compartment of hippocampal neurons and thus modulates its synaptic plasticity (Canal et al., 2011). When there is little ERK1/2 activity then KSR1 is concentrated in the dendritic spines. With neuronal activity there is a consequential increase in ERK1/2 activation and KSR translocates to the dendrite as a result of feedback phosphorylation which regulates the signaling output of MAPK. Importantly, a feedback impaired variant of KSR1 shows that ERK-mediated feedback phosphorylation of KSR1 prevents sustained ERK signaling in neurons and thus may contribute to signaling homeostasis and also to homeostasis of synapses (Canal et al., 2011). KSR preferentially acts on MAPK signals originating from cholesterol rich domains of the plasma membrane that further highlights their spatial regulation of ERK signals (Matheny et al., 2004).

KSR1 can also route signals to the cytoplasm: in addition to binding Raf and MEK, KSR1 (but also other scaffolds such as Sef and IQGAP1) helps to assemble ERK1/2 dimers that are essential for interaction of ERK with cytoplasmic substrates (Casar et al., 2008). For example, large amounts of cPLA_2_, a cytoplasmic substrate of ERK1/2, were associated to KSR1 following EGF stimulation. As simultaneous binding of cPLA_2_ and the scaffold to ERK2 would be sterically unlikely and since ERK is known to homodimerize upon activation, the current model is that ERK dimers are formed where one molecule of ERK2 is bound to cPLA_2_ whilst the other is bound to KSR1. The dimerization mechanism occurs when a phosphorylated ERK monomer bound to scaffold interacts with a free phospho-ERK monomer that has probably been released from a MEK-ERK complex. These dimers specifically phosphorylate ERK substrates in the cytoplasm. Alternative routes for the phosphorylated ERK monomers have been suggested including translocation to the nucleus to phosphorylate transcription factors or dimerizing freely in the cytoplasm and then moving to the nucleus as Casar et al. also found a few ERK dimers in the nucleus following activation (Casar et al., 2008). Therefore, KSR1 can control whether substrates in the nucleus or cytoplasm are activated. The roles of scaffolds such as KSR1 may be very different in different cell types, and the cell-type and tissue-specific expression of scaffolds may play an important role to attribute different functions to MAPK signaling in different tissues (see Figure 6).

## Scaffolds and ultrasensitivity

Cellular decision-making involves the ability to respond to a continuously variable stimulus with a defined answer, either yes or no. Famous all or none switches (biological responses) are the Xenopus oocyte maturation in response to progesterone (Ferrell and Machleder, 1998) or shmooing of yeast cells in response to a/α-factor (Paliwal et al., 2007; Malleshaiah et al., 2010). The reason for switch-like behavior may be a steep stimulus-response profile, described as ultrasensitivity, but it might also be bistability where the system switches from one stable steady state to another. Ultrasensitive responses are beneficial since they allow to filter out noise and to respond vigorously to appropriate stimuli. Supposedly, ultrasensitive responses are generated by mechanisms that are embedded in the signaling network structure and might be visible already at the level of the respective MAPK. For example, the response of JNK is ultrasensitive when stimulating either HeLa, HEK 293, or Jurkat T Cells with sorbitol or anisomycin (Bagowski et al., 2003). In general, multistep processes of (de)activation can generate robust ultrasensitive responses (Deshaies and Ferrell, 2001; Blüthgen and Herzel, 2003; Salazar and Höfer, 2009). Examples are the activation of a kinase by several consecutive phosphorylation events, but also saturation of an enzyme by stepwise binding of ligands with positive cooperativity. Positive feedbacks can create bistability, however it was shown that a system with distributive multisite (de)phosphorylation does not need additional regulatory layers for bistability to exist (Markevich et al., 2004). With the perception of scaffolding molecules as a vital part of MAPK signaling cascades, several experimental and theoretical studies have focused on the question whether scaffolds contribute positively or negatively to ultrasensitivity.

In theory, the three-tiered MAPK cascade comprising single phosphorylation of Raf and dual phosphorylation of MEK and ERK, is inherently ultrasensitive (Huang and Ferrell, 1996) and a critical determinant of this property is the assumed distributive mechanism of (de)phosphorylation. Levchenko et al. studied the effect of a generic scaffold molecule that sequesters a substrate and its kinase and assumed that the scaffold would contribute to processive phosphorylation (Levchenko et al., 2000). This assumption dates back to an experimental study, where a so far unidentified protein enhanced dual phosphorylation of ERK by MEK (Scott et al., 1995). Processive rather than distributive phosphorylation would then counteract ultrasensitivity. Not surprisingly, the model predicts that the stimulus-response relationship of the cascade becomes more graded at increasing levels of supporting scaffold. However, to date experimental proof is still lacking that would support the function of scaffolds to promote processive phosphorylation. The dynamics of ERK phosphorylation in EGF-stimulated HeLa cells is in accordance with processive phosphorylation, but a knockdown of either KSR, MP-1, IQGAP1, Paxillin, or β-arrestin 1/2 did not change processivity but only the ERK phosphorylation efficiency (Aoki et al., 2011). Another theoretical study highlights that even when rapid enzyme-substrate rebinding is possible the necessity of exchanging ADP for ATP on the kinase might preclude this opportunity for quasi-processive phosphorylation (Takahashi et al., 2010).

In general, a gradual progress of morphological changes can be observed when yeast cells are stimulated with increasing amounts of pheromone (Paliwal et al., 2007). In accordance with these gradual changes in phenotype, is the gradual expression of a Fus1-GFP reporter in response to pheromone which was observed in single yeast cells (Poritz et al., 2001). The graded response profile is neither established by the action of negative feedback regulators, nor by the sole presence of the scaffold Ste5 (Takahashi and Pryciak, 2008). Once signaling to Fus3 is decoupled from Ste5 membrane translocation by direct constitutive activation of Ste11, signaling becomes ultrasensitive. This confirms a switch-like behavior of the isolated MAP3K-MAP2K-MAPK module, even in the presence of a scaffold and suggests that the graded response is due to Ste5 membrane localization upon pathway stimulation. Also here it is speculated that the common view of a fully occupied scaffold, in which all bound kinases influence each other in a beneficial way, i.e., by allowing processive activation, might be wrong. Ultimately, the graded response might be a result of the close proximity of differently occupied scaffolds due to membrane localization. The kinases that sit on different scaffolds then can activate each other in *trans* (Takahashi and Pryciak, 2008) (see Figure 4B). Interestingly, a theoretical model confirms that at constitutive Ste11 activity (in the absence of pheromone stimulation) an increased ultrasensitivity can only be explained by *trans*-activation of kinases that are located on different scaffolds and whose interactions are characterized by weak affinities (Thalhauser and Komarova, 2010).

Despite the gradual morphological changes of yeast in response to increasing levels of pheromone, the final decision to form a shmoo is switch-like. In an experimental analysis the average response of a population of yeast cells on the level of Fus3 appeared to be slightly ultrasensitive with a Hill coefficient n_H_ ~2.2 (Hao et al., 2008). A mathematical model of the pheromone response can reproduce this Hill coefficient, however, the model suggests that ultrasensitivity of the response is generated at a level upstream from Ste5-scaffolded signaling (Thalhauser and Komarova, 2010). In more detail, the model incorporates a dual influence of pathway activation as it contains a stimulus-dependent rate of proper Ste7-alignment (by membrane localization) and a stimulus-dependent rate of Ste11-activation. Increasing either the rate of membrane-binding or the rate of Ste7-alignment, the system response can be tuned to become more graded (Thalhauser and Komarova, 2010).

In contrast, Malleshaiah et al. attribute an active role to the scaffold in producing the all-or-none shmoo response (Malleshaiah et al., 2010), as the dissociation of the Ste5-Fus3 complex in response to pheromone stimulation is highly ultrasensitive. Strikingly, the response of a Fus3-docking impaired Ste5 mutant becomes graded. At the basis of the steep response is a competition of the alpha-factor-induced phosphatase Ptc1 with Fus3 for access to several distinct phosphorylation sites on the scaffold. Progressive dephosphorylation of these sites negatively influences the binding affinity of the Ste5-Fus3 complex (Malleshaiah et al., 2010).

Graded stimulus-response profiles are frequently observed in mammalian cells (Whitehurst et al., 2004; MacKeigan et al., 2005; Tian et al., 2007). However, experimental data also suggest that the properties of the signal-response profile are not determined on the level of the scaffold. For example, single cell measurements of ERK phosphorylation in Jurkat cells show that the MAPK pathway may either show a graded response to chemokine SDF-1 stimulation or a digital response when the T-cell receptor is involved. The response pattern of both pathways is not influenced by the expression level of KSR1 (Lin et al., 2009). In baby hamster kidney cells the response to EGF appears graded. EGF-signaling is initiated at Ras-GTP nano-clusters that consist of roughly 7 Ras-GTP molecules with a lifetime of about 0.4 s and which recruit Raf to the membrane. Each nano-cluster is shown to act as a molecular switch, that converts a graded input to an all or none response (Tian et al., 2007). However, as the amount of clusters is increasing linearly with the stimulus, the integrated response over all clusters is graded, yielding a linear response of ERK phosphorylation to EGF stimulation.

In summary, contrary to previous reports switch-like responses seem to be possible even in the presence of a scaffold molecule (Levchenko et al., 2000). A theoretical study even finds that the presence of a scaffold can increase the likelihood of bistability to occur, especially when the affinity of substrate and modifying enzyme is low. Parameter sets that support bistability are characterized by a limiting concentration of scaffold compared to the level of substrate that is modified on the scaffold and a scaffold-substrate dissociation constant that varies with substrate phosphorylation state (Chan et al., 2012). On the other hand, when the response of a pathway is graded, this is not necessarily due to the presence of a scaffold, but may be due to strong negative feedbacks that are known to determine the pathway's behavior (Sauro and Kholodenko, 2004; Cirit et al., 2010; Sturm et al., 2010; Fritsche-Guenther et al., 2011), or under some situations ultrasensitivity might be enhanced by positive feedbacks (Santos et al., 2007).

To date, the discussion about stimulus-response profiles suffers from ambiguities that result from measurement of responses at different levels (MAPK, MAPK-target-reporter gene, morphological response), evaluation of the response in different ways (integrated response or response at a certain time after stimulation) and evaluation of the response at the single cell or population level. Ultrasensitivity at the single-cell response profile is potentially lost when averaging over a population of cells. For example, the response of JNK to progesterone in Xenopus oocytes is ultrasensitive at the single cell level. However, as the critical concentration for a switch to occur varies greatly between single oocytes, the JNK response of a population of cells appears to be graded (Bagowski and Ferrell, 2001). Thus, the precise role of scaffolds on changing the ultrasensitivity of stimulus response has yet to be established.

## Conclusions

While more and more mechanistic details about the action of scaffolds emerge, the role for scaffolds on the dynamics of MAPK signaling is currently not well-established for mammalian cells. Although many recent studies show how KSR1 controls spatial routing of signals and facilitates signal initiation, we still lack a comprehensive understanding of the role of the scaffold. Detailed mechanistic mathematical modeling and quantitative experimentation has been useful in the past by shedding light on how signaling works dynamically. For the yeast scaffold Ste5, mathematical models to explore the action of the scaffold have come to some maturity and provide detailed understanding of the role of this scaffold in this pathway. Similarly, we think that it would be timely to quantitatively study the role of KSR1 on signaling dynamics in mammalian cells, with detailed quantitative experiments that estimate the concentrations of scaffolds and signaling molecules. While the rather abstract models of scaffolds in mammalian MAPK signaling were helpful to conceptualize how scaffolds act, they also led to misleading results: The commonly accepted notion of the presence of an optimal scaffold concentration has led to the conclusion that scaffolded signaling might be more sensitive to perturbations in the concentration of pathway components (Ferrell, 2000). However, for this conclusion the details matter, and the behavior changes when simulating a scaffold model with realistic concentrations for signaling molecules. Since the ligands of the scaffold show different expression levels, a large range of scaffold concentrations may optimally support signaling.

Working toward realistic models for scaffolds is challenging due to combinatorial explosion: a comprehensive model of signaling via Ste5 which involves all 27 possible scaffold configurations both in cytoplasm and when located to the membrane exemplifies this complexity (Shao et al., 2006). Taking into account the oligomerization of Ste5 the number of signaling complexes would increase to 27 × 27 = 729. Thus, simpifying assumptions will be needed to develop these models and parameterize them.

Differential expression of scaffolds in different cell types may be one of the main reasons why many cell-type-specific interactions and feedbacks exist. Hence, once we know more about how scaffolds shape signal transduction, we will most likely also understand how and why MAPK signaling gains different roles in different tissues. Again, one can learn from yeast, where the scaffold protein Ste5 has been modified using a synthetic biology approach to recruit molecules that are either positive or negative regulators. These regulators are either induced by the pathway or expressed constitutively, either alone or in combination, which affects the shape of the pathway response in a predictable manner. This study highlights not only a chance for potential therapeutic results by targeted engineering of a pathway but also documents the various functions a scaffold might bear within the cell (Bashor et al., 2008).

### Conflict of interest statement

The authors declare that the research was conducted in the absence of any commercial or financial relationships that could be construed as a potential conflict of interest.

## Research Topic:

From structural to molecular systems biology: experimental and computational approaches to unravel mechanisms of kinase activity regulation in cancer and neurodegeneration

## Topic Editor:

Matteo Barberis, Humboldt University Berlin, Germany; Max Planck Institute for Molecular Genetics, Berlin, Germany
